# How teacher behaviour in student‐teacher dyads relates to boys' and girls' mathematics anxiety. An investigation of interpersonal profiles

**DOI:** 10.1111/bjep.70104

**Published:** 2026-06-26

**Authors:** M. Frühauf, J. M. Etzel, B. Hannover

**Affiliations:** ^1^ Department of Education and Psychology Freie Universität Berlin Berlin Germany; ^2^ Department of Psychological Diagnostics, Differential Psychology, and Psychological Methods Charlotte Fresenius University‐ University of Psychology Hamburg Germany

**Keywords:** circumplex, gender, interpersonal behaviour, mathematics anxiety, self‐concept, student‐teacher‐relationship, teacher‐expectations

## Abstract

**Background & Aims:**

Mathematics anxiety impairs student learning, particularly affecting girls. Grounded in interpersonal theory, this study investigated how interpersonal teacher behaviours in student‐teacher dyads relate to boys' and girls' mathematics anxiety. We defined these behaviours using an interpersonal circumplex, which blends agency (guidance, control) and communion (warmth, sensitivity). Additionally, we examined whether these relationships are indirectly linked through students' mathematics self‐concept and their perception of teacher expectations.

**Sample:**

We surveyed 42 mathematics teachers (32 female) and 743 of their primary school students (362 female).

**Methods:**

Teachers described their interpersonal teacher behaviour toward each individual student in their class. Using circumplex modelling, we derived teacher agency and communion as underlying dimensions of teachers' interpersonal profiles. We then applied structural equation modelling to analyse which profiles most strongly relate to girls' and boys' mathematics anxiety and to test for indirect associations.

**Results:**

Teacher behaviour combining high agency with low communion was associated with higher mathematics anxiety in the student‐teacher dyad. Low teacher communion was significantly related to mathematics anxiety in girls only. Indirect associations between teacher behaviour and student mathematics anxiety via self‐concept and student‐perceived teacher‐expectation emerged for agency in both genders, but for communion only in girls.

**Conclusions:**

Lower teacher agency in dyadic interactions may be linked to a less anxious approach to mathematics. High teacher communion may be particularly vital for girls as they are more likely to form strong bonds with their teacher than boys and often confront negative performance‐related stereotypes in mathematics.

## INTRODUCTION

Mathematics (math) anxiety detrimentally affects student learning. For instance, PISA 2022 data indicate that individual differences in math anxiety account for approximately 25% of the variance in student math performance between countries (OECD, 2023). A meta‐analysis on 62 studies by Pelegrina et al. (2025) found that prior math anxiety predicted subsequent math performance (and vice versa). Preventing math anxiety is therefore essential to facilitate students' acquisition of mathematical skills (Barroso et al., 2021; Hembree, 1990). Research demonstrates that contextual factors significantly influence math anxiety, with teachers playing a crucial role in shaping the supportiveness of the learning environment (Gunderson et al., 2012; Wang et al., 2021). In a comprehensive review, O'Hara et al. (2022) identified how learning environment characteristics relate to student math anxiety. While behaviours such as classroom activation, clear goal structures, strong diagnostic skills and teacher support can reduce anxiety, other behaviours—such as the use of fear or punishment—can amplify it.

Our study utilizes a theoretical paradigm that integrates these various teacher behaviours into two basic dimensions of interpersonal behaviour. Specifically, we relied on interpersonal theory (Horowitz & Strack, 2011; Leary, 1957), which describes dyadic interactions on two dimensions: communion (e.g., warmth, sensitivity vs. coldness, aloofness) and agency (e.g., direction, guidance vs. submissiveness, passivity). Together, these two orthogonal dimensions span the interpersonal circumplex (Figure 1), a model that describes interpersonal behaviour using eight prototypical types (octants). Each octant encompasses a different combination of agency and communion. This approach allowed us to identify which octants are most strongly related to student math anxiety. Furthermore, we extended this framework to include potential mediating pathways. Specifically, we examined, first, whether interpersonal teacher behaviour is indirectly associated with math anxiety through student‐perceived teacher‐expectations and math self‐concept. Second, we investigated whether these pathways differ for boys and girls.

**FIGURE 1:**
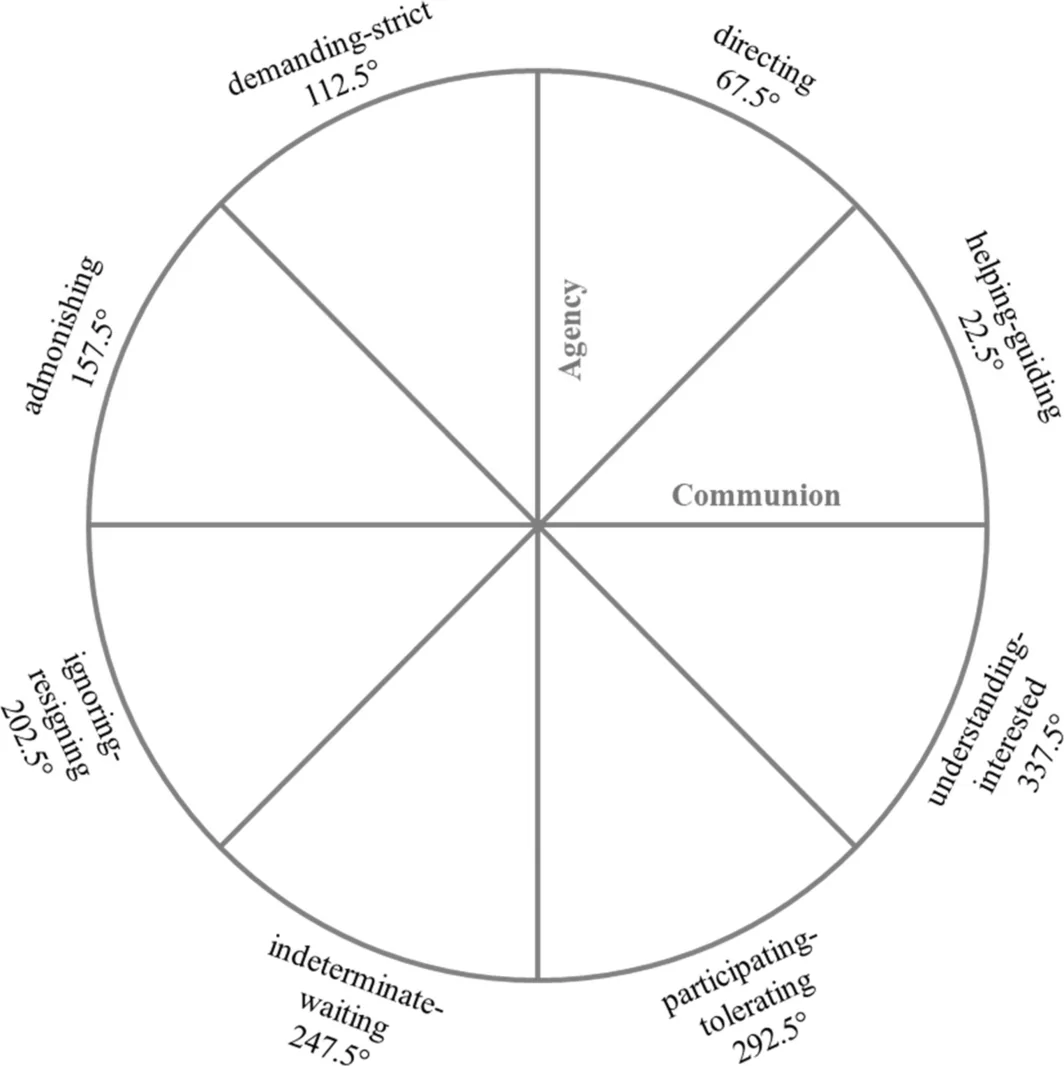
Theoretical circumplex model of interpersonal teacher behaviour.

O'Hara et al. (2022) noted that most studies included in their review investigated the relations between interpersonal teacher behaviour and student math anxiety by relying exclusively on student reports. This approach is problematic because a student's anxiety can bias their perception, leading to inaccurate evaluations of actual teacher behaviour. To address this, our study utilized two different data sources: teachers described their behaviour toward each student, while students reported their own math anxiety.

Interpersonal theory (Horowitz & Strack, 2011; Leary, 1957) emphasizes that behaviour in dyadic interactions is interdependent: the same teacher may behave differently depending on the individual student's characteristics and behaviours (Domen et al., 2020; Frühauf et al., 2025; Kreutzmann et al., 2024). Consequently, we measured teachers' behaviour at the dyadic level, focusing on interactions with individual students rather than general classroom behaviour.

While previous research on math anxiety has focused on grade levels 9 to 12 (O'Hara et al., 2022), we deliberately targeted primary school students. This age group represents a critical formative period for building foundational math skills—in particular, a basic understanding of numbers and arithmetic operations, both of which are prerequisites for future academic success in math (National Research Council, 2001). Concurrently, the primary school years are a phase in which teachers' interpersonal behaviours heavily relate to students' social, motivational and emotional development (Jerome et al., 2009; Valiente et al., 2019). For instance, Kreutzmann et al. (2026) showed that teachers' interpersonal behaviour is associated with students' peer networks, including students' choices of whom to cooperate with in class (for a meta‐analysis on teachers' influence on peer relations, Endedijk et al., 2022). Similarly, Koeppen et al. (2025) observed that students' need satisfaction and motivation depend on the interpersonal behaviour teachers display toward them in dyadic interactions. Furthermore, primary school students rely on feedback from their teachers to build domain‐specific self‐esteem, self‐concepts and control beliefs. Indeed, Hattie's (2023) meta‐analytic findings provide strong evidence that primary school students are particularly responsive to teacher feedback, with this developmental stage showing the highest effect sizes. During these early years, self‐beliefs are still highly malleable and susceptible to external influences (Dapp & Orth, 2024), such as teacher behaviour. Hence, primary school students are at a developmental phase where their academic and emotional trajectories are uniquely sensitive to their teacher's interpersonal behaviour—a period that may also coincide with the very onset of math anxiety (Pelegrina et al., 2025). Investigating this timeframe, therefore, provides insight into a critical window during which negative emotions toward math may become consolidated and resistant to change over time.

### Math anxiety in girls and boys

Math anxiety involves feelings of fear, dread, apprehension or tension when engaging with math (Ashcraft, 2002) and is reciprocally linked to performance deficits (Barroso et al., 2021; Pelegrina et al., 2025). Math‐anxious students tend to avoid math (Choe et al., 2019) and show lower engagement during math lessons (Hembree, 1990; Ramirez et al., 2018). Because math anxiety is already present in primary school (Ramirez et al., 2013; Vukovic et al., 2013), early intervention is crucial—especially for vulnerable groups.

Girls represent a particularly vulnerable group, as negative gender stereotypes regarding math competence persist (Henschel et al., 2023; Nosek et al., 2002). Math remains more closely associated with anxiety and negative attitudes in girls than in boys (Vos et al., 2023), regardless of actual performance (Geary et al., 2023). Notably, even though gender gaps in math achievement have largely disappeared (Else‐Quest et al., 2010), girls continue to report higher levels of math anxiety.

### Using the interpersonal circumplex to describe teachers' behaviour profiles

Teacher behaviour encompasses both social–emotional and instructional aspects (for an overview, see Opdenakker, 2023). Similarly, interpersonal theory (e.g., Horowitz & Strack, 2011; Leary, 1957; Wright et al., 2023) posits that all interpersonal behaviour can be mapped onto two orthogonal dimensions: communion and agency. Communion involves behaviours essential for establishing and maintaining social connections, such as warmth, friendliness, sensitivity and emotional responsiveness. In educational settings, communion reflects social–emotional support, capturing how warmly and sensitively a teacher interacts with a student. Agency describes behaviours directed toward goal achievement, including dominance, guidance, control and competence (Horowitz & Strack, 2011; Leary, 1957). In the classroom, agency manifests as instructional support, representing how effectively the teacher guides and monitors an individual student's learning progress.

Traditionally, these two dimensions have been studied in isolation: social–emotional support (communion) is typically examined within attachment theory (Ahnert et al., 2006; Pianta, 1999), whereas instructional support (agency) is prominent in research on teaching effectiveness (Alp Christ et al., 2022) and adaptive teaching (Bernard et al., 2019).

In the present study, we simultaneously modelled both dimensions of interpersonal teacher behaviour to capture their distinct relations with student math anxiety. This simultaneous approach is essential because interpersonal theory posits that all different types of interpersonal (teacher) behaviour are fully described by the interpersonal circumplex, which is spanned by the two orthogonal dimensions of agency and communion (Figure 1). As illustrated in Figure 1, the interpersonal circumplex is divided into eight octants, each representing a specific weighted combination of agency and communion. For example, the “demanding‐strict” octant combines low communion with high agency (for an overview of all octants, see Table S1). Consequently, demanding‐strict teacher behaviour places less emphasis on the student's individual interests and needs, focusing instead on providing precise instructions, monitoring behaviour and correcting it when necessary.

The interpersonal circumplex illustrates why agency and communion must be considered together when investigating student math anxiety. Because different octants represent uniquely weighted blends of these two dimensions, focusing on only one axis at a time may oversimplify dyadic interactions. For example, the teacher's close monitoring of learning activities (strong agency) may be perceived as either supportive or controlling by the student, depending on whether the teacher is affectionate and warm (strong communion) or suspicious and dismissive (weak communion). Conversely, the teacher's attentiveness (strong communion) can yield different outcomes for the student depending on the level of agency: it may manifest as granting autonomy (weak agency) or as overprotective control (strong agency). Therefore, examining only one dimension at a time fails to capture the multifaceted nature of interpersonal teacher behaviour and does not do justice to the established circumplex model.

The circumplex structure also has several important implications for measuring and analysing interpersonal teacher behaviour and its relationships with external variables (Etzel, 2025; Gurtman & Pincus, 2003). Firstly, the model implies that adjacent octants (e.g., “demanding‐strict”, “directing”) are more similar than octants that are further apart. Opposing octants (e.g., “demanding‐strict”, “participating‐tolerating”) are assumed to be most dissimilar. Consequently, valid assessment instruments based on this model must exhibit a correlation structure that reflects this cosine‐shaped pattern. Secondly, individual teacher profiles across all octants (typically assessed as a vector of eight scale scores for each individual) tend to align with this circular structure (Gurtman & Balakrishnan, 1998). For instance, teachers who score high on “demanding‐strict” should also score highly on the adjacent octants “directing” and “admonishing”, but low on the opposing “participating‐tolerating” octant.

Thirdly, and most importantly, the circumplex can summarize how external variables—such as math anxiety—relate to interpersonal profiles (Gurtman, 1992). The correlations between the eight octant scales and math anxiety are expected to follow the same circular pattern, meaning that the adjacent octants show similar associations with math anxiety, while opposing octants show contrasting ones. In our analytic approach, the relationship between interpersonal teacher behaviour and student math anxiety is visualized as a single vector within the circumplex space. In this representation, the vector's angular orientation indicates the exact blend of communion and agency that is most strongly associated with student math anxiety, while the vector's length indicates the magnitude of the relationship (Gurtman & Pincus, 2003).

### Interpersonal teacher behaviour and student math anxiety in student‐teacher dyads

In educational psychology, the interpersonal circumplex is widely used to describe teachers' general teaching styles (Aelterman & Vansteenkiste, 2023; Wubbels et al., 1993; Wubbels & Brekelmans, 2005). However, while teaching styles capture a teacher's general approach toward the whole class, actual teacher behaviour often varies from student to student (Frühauf et al., 2025; Koeppen et al., 2025). We argue that a teacher's behaviour during one‐on‐one interactions is meaningfully related to an individual student's emotional experience in math. Therefore, our research focused specifically on the microsystem of the student‐teacher dyad.

Within the student‐teacher dyad, we expected that teacher behaviour combining high communion and moderate‐to‐low agency (i.e., “understanding‐interested” and “participating‐tolerating” behaviour, Figure 1) would relate most strongly to low student math anxiety. Such warm, sensitive and autonomy‐supportive behaviours provide emotional and academic reassurance during potentially distressing situations in the classroom (Goetz et al., 2021; Van Vemde et al., 2022; Zee & Roorda, 2018). Conversely, we expected that opposing teacher behaviours—characterized by low communion and high agency (“demanding‐strict” and “admonishing”; see Figure 1)—would be associated with elevated math anxiety: teachers who lack communion fail to offer emotional support in stressful situations, such as when a student struggles to comprehend a math problem or fears solving one at the blackboard (Emslander et al., 2025). These expectations are substantiated by research showing that high teacher agency restricts students' sense of autonomy and competence (Deci & Ryan, 2000), thwarting high‐quality forms of motivation, need satisfaction and positive affect (Aelterman & Vansteenkiste, 2023; Koeppen et al., 2025; Pekrun, 2006).

Further evidence for our assumptions comes from classroom‐level research, which indicates that strongly communal and weakly agentic teacher behaviour is related to lower student math anxiety. For instance, teacher sensitivity (Aldrup et al., 2020) and strong teacher support (Ahmed et al., 2010; Li et al., 2023) consistently coincide with weaker classroom‐level math anxiety. Conversely, teaching practices characterized by fear, punishment or competition are linked with heightened math anxiety at the classroom level (Frenzel et al., 2007; Putwain & Symes, 2011; Westphal et al., 2018). These findings align with our expectation that high teacher communion coincides with weaker math anxiety in the student‐teacher dyad.

Similarly, structural aspects of teaching show distinct associations with math anxiety. While clear, structured instruction (Frenzel et al., 2007) and classroom activation (Lazarides & Buchholz, 2019) are associated with lower classroom‐level math anxiety, controlling teacher behaviour is linked to heightened math anxiety levels within the classroom (Assor et al., 2005). This aligns with our hypothesis that, within the dyad, lower teacher agency is associated with reduced math anxiety. In the current study, we utilized the interpersonal circumplex to integrate these scattered findings regarding math anxiety within an established, comprehensive framework of interpersonal behaviour.

### Student perception of teacher behaviour in student‐teacher dyads

In our study, we linked teacher‐reported interpersonal behaviour to student‐reported math anxiety. To capture the student's perception of the teacher, we additionally examined student‐perceived teacher expectations regarding their math competence. A teacher's perception of a student's ability often manifests in their interactions (Eccles & Wigfield, 2020). Against this background, we expected that low teacher expectations would translate into less warm and sensitive interactions (low communion) and a tendency to strongly direct and control the student's learning (high agency). The student, in turn, may interpret this teacher behaviour as a lack of acceptance and a sign that the teacher has little confidence in their abilities (Gentrup et al., 2020). Finally, we assessed the student's self‐concept in math, which represents a critical predictor of math anxiety (Pekrun, 2006; Pekrun et al., 2023).

## THE PRESENT INVESTIGATION

The current study aimed, first, to examine the relationships between dyadic interpersonal teacher behaviour and primary school students' math anxiety. Second, we investigated whether these relationships are indirectly associated through students' cognitive appraisals (perceived teacher‐expectation, self‐concept). Third, we explored whether these pathways differ for boys and girls.

We derived dyadic interpersonal teacher behaviour profiles by calculating scores for the agency and communion axes, examining their relationship with students' math anxiety and appraisals. This approach allowed us to project these relationships as vectors onto the interpersonal circumplex, thereby identifying the specific behaviour profiles most strongly associated with students' math anxiety.

We expected that interpersonal teacher behaviour combining low communion and high agency would show the strongest association with student math anxiety (Hypothesis 1). Empirically, this hypothesis would be supported by a significant association between math anxiety and the circumplex of interpersonal teacher behaviour (Nagy et al., 2019), yielding a vector whose angular orientation points toward the “demanding‐strict” and “admonishing” octants (Figure 1).

Furthermore, we assumed that teacher behaviour characterized by low communion and high agency would be associated with (a) lower student‐perceived teacher expectations, (b) a lower math self‐concept and (c) higher math anxiety. Hence, we expected that the relationship between student math anxiety and teacher agency (Hypothesis 2a) and teacher communion (Hypothesis 2b) would be indirectly associated through student‐perceived teacher expectations. Similarly, we expected that the relationship between student math anxiety and teacher agency (Hypothesis 3a) and teacher communion (Hypothesis 3b) would be indirectly associated through student math self‐concept. For exploratory purposes, all analyses were also conducted separately for girls and boys.

## METHOD

### Sample and procedure

We surveyed 42 (32 female) math teachers (age: 43.2 years, *SD* = 12.7; teaching experience: 13.4 years, *SD* = 11.2) and their respective classes from grades 3–6 from 13 primary schools in Berlin, Germany. Teachers described their behaviour toward each student in their classroom (on average 22.9 (*SD* = 2.4) students per teacher), yielding 957 dyadic interpersonal teacher behaviour descriptions. Of the students, 743 (mean age 10.1 years, *SD* = 1.3) completed the classroom survey after their parents provided informed consent. Within this sample, 362 students identified as female (48.7%), 379 as male (51.0%) and two as diverse. As a proxy for a potential migration background, we asked students to indicate the language(s) they were raised with. While 59.1% of all students were raised exclusively with German, the remaining 40.9% were raised with either German and another language or exclusively with a non‐German language during their early childhood. This latter group was classified as coming from immigrant families.

Data collection was part of a research project (Qualität schafft Chancen‐Konsortium, 2025) funded by the German Research Foundation (HA 2381/17‐1). Approval was obtained from both the School Senator Berlin and the ethics committee of the Department of Education and Psychology of the Freie Universität Berlin (025/2021). Participation was incentivized with 50 Euros per school class and 100 Euros per teacher.

### Measures

#### Interpersonal teacher behaviour

Teachers described their interpersonal behaviour toward each student using the 18‐item version of the Questionnaire on Dyadic Interpersonal Teacher Behaviour (DITeB; Kreutzmann et al., 2024). Each octant (Figure 1) is represented by two to three items. For instance, the octant “helping‐guiding” includes items such as: “If [unique identifier] has difficulties solving a task, I am happy to show them step by step how to solve the problem.” (for the full item set, see Kreutzmann et al., 2024). Teachers described their behaviour toward each student sequentially on 7‐point Likert scales (1 = *strongly disagree*, 7 = *strongly agree*). We computed scale means for each of the octant scales (McDonald's ω estimates ranged from .65 to .86). These scales were then used to derive scores on the agency and communion dimensions for each dyad (see Statistical Analysis).

#### Student math anxiety

Students reported their math anxiety on an 8‐item scale adapted from Henschel and Roick (2020; e.g., “I worry that the problems in my math book are too difficult for me.”, or “I am nervous when my math teacher asks me something in math class.”; 1 = *strongly disagree* to 4 = *strongly agree*; reliability McDonald's *ω* = .89).^1^

#### Student perceived teacher‐expectation

We used 5 items from a scale by Gärtner (2010; e.g., “My math teacher thinks that math is easy for me”, or “My math teacher has a lot of confidence in me during my math lessons”; 1 = *strongly disagree* to 4 = *strongly agree*; reliability McDonald's *ω* = .89).

#### Student math self‐concept

We used a 3‐item subscale from the German version (Arens et al., 2011) of the Self‐Description Questionnaire‐I (SDQ‐I; Marsh, 1990; e.g., “I am good at math”, or “Math is easy for me”; 1 = *strongly disagree* to 4 = *strongly agree*; reliability McDonald's *ω* = .91).

#### Covariates

As students' age and math competence may be associated with students' math anxiety and interpersonal teacher behaviour (Barroso et al., 2021), these variables were included as covariates in all models. We assessed math competence (numeric reasoning skills) with standardized tests (for grades 3 to 4: BEFKI 3‐4; Schroeders et al., in preparation; for grades 5 to 7: BEFKI 5‐7; Schroeders et al., 2020). Students answered 16 single‐choice items with four options. To complete the test, third and fourth graders were given 18 min, whereas fifth and sixth graders had 14 min to complete the task. The tests were scaled using a one‐parameter logistic IRT model estimated with the R‐package *eatModel* (R Core Team, 2021; Weirich & Hecht, 2018). We calculated Weighted Likelihood Estimates (WLE; Warm, 1989) as estimates of student ability (WLE reliability was r_wle_ = .76). Further, to account for students' overall emotional experience in school, we controlled for general school satisfaction (Stanat et al., 2018; 3 items, e.g., “I enjoy going to school”, 4‐point Likert‐scale; *α* = .81).

### Statistical analysis

We used M*plus* 8.8 (Muthén & Muthén, 2017) for all analyses unless stated otherwise. We accounted for the nested data structure by estimating cluster‐robust standard errors (for additional information, see Note S3).

#### Circumplex structure

We used the circular stochastic process model (SPMC; Browne, 1992; Nagy et al., 2019) to examine the structural validity (i.e., the fit of the circumplex structure) of the interpersonal teacher behaviour measure. The SPMC analyzes the covariance structure of the interpersonal teacher behaviour octant scales. Crucially, it tests whether the octant scales exhibit the expected structural pattern at the level of common scores by modelling a latent circumplex. In other words, it tests whether adjacent octants (e.g., “helping‐guiding” and “understanding‐interested”) are more strongly correlated than octants that are further apart (e.g., “admonishing” and “understanding‐interested”). Ensuring the structural validity of a measure is an essential step before deriving profile scores for each student‐teacher dyad. This is because the validity of these intraindividual profiles rests on the validity of the structural model at the interindividual level (e.g., Nagy et al., 2009). Due to its high flexibility, the SPMC allows researchers to relax the strict assumption of perfectly equidistant scale positions around the circumplex, thereby testing a quasi‐circumplex (Guttman, 1954). Specifically, the SPMC estimates the internal correlation structure of the octant scales using a Fourier series approximation. The central model parameters are: (1) the shape of the circumplex's underlying correlation function (the number of Fourier components), (2) the proportion of variance attributable to profile levels and profile patterns, (3) the angular locations of the octant scales around the circle ($\hat{\theta }$_*j*_) and (4) the scales' communalities $\hat{h},$ which represent the proportion of their variance shared with the latent circumplex. A detailed and more technical description of the SPMC can be found in the supplemental material (Note S1).

#### Relationships between interpersonal teacher behaviour and student math anxiety

For the main analyses, we first derived agency and communion scores from the eight interpersonal teacher behaviour subscales (Gurtman & Pincus, 2003). These scores are calculated as linear combinations of the eight interpersonal teacher behaviour scale scores using the sine $\left({\text{Agency}}_{j}=\frac{1}{4}\;\sum_{i=1}^{8}\left(\sin\;\left({\hat{\theta }}_{i}\right)S_{j,i}\right)\right)$ and cosine $\left({\text{Communion}}_{j}=\frac{1}{4}\;\sum_{i=1}^{8}\left(\cos\left({\hat{\theta }}_{i}\right)S_{j,i}\right)\right)$ of the estimated angular locations from the fitted SPMC models (${\hat{\theta }}_{i}$) as weights. The two scores represent the dyads' Cartesian coordinates in the circumplex space, summarizing a teacher's interpersonal profile toward the student. Using SEM, we then regressed student math anxiety on agency and communion (Model 1). Subsequently, we added student perceived teacher‐expectation and math self‐concept to test indirect associations (Model 2). Student math anxiety, perceived teacher‐expectation and self‐concept were incorporated as latent variables (Figure S1). We used robust maximum likelihood (MLR) estimation to account for non‐normality and handled missing values via full information maximum likelihood (FIML). The standardized path estimates (β_AG_ and β_CO_) of Model 1 can be used to visualize the relationship between interpersonal teacher behaviour and math anxiety as the endpoints of a vector in the interpersonal circumplex. The angular orientation of the vector $\left(\delta ,=,\tan^{-1},\left(\frac{\beta_{\mathrm{AG}}}{\beta_{\mathrm{CO}}}\right),✕,\frac{180}{\pi }\right)$ corresponds to the interpersonal teacher behaviour most highly correlated with math anxiety, while the vector length $\left(\mathrm{VL}=\sqrt{{\beta_{\mathrm{AG}}}^{2}+{\beta_{\mathrm{CO}}}^{2}}\right)$ indicates the strength of the association (Gurtman & Pincus, 2003). We employed multiple‐group analysis to estimate and compare path estimates for boys and girls. This allowed us to inspect potential gender differences in the relationship between interpersonal teacher behaviour and student math anxiety. Additionally, this approach enabled the estimation of gender‐specific vectors to visualize the relationship between interpersonal teacher behaviour and math anxiety in boys and girls separately.

### Transparency and openness

Study materials, analysis syntaxes and data are available at https://osf.io/pcfbk.

## RESULTS

### Fit to the structural models

First, we tested the interpersonal teacher behaviour scale's fit to the circumplex model. Table S3 shows that the model with *m* = 2 components in the Fourier series provided the best fit, *χ* ^2^ (11) = 32.77, *p* < .001, RMSEA = .046, CFI = .958, SRMR = .049. Overall, the configuration of interpersonal teacher behaviour octants reflected a quasi‐circumplex that was very close to the theoretically assumed model (Table 1, Fisher's A* > 92, Fisher et al., 1985).

**TABLE 1:** Results of the SPMC model.

	θ_target_	$\hat{\theta }$ (*SE*)	$\left|\Delta \hat{\theta }\right|$	A*	$\hat{h}$
1. Helping‐guiding	22.5°	17.64° (9.21°)	4.86°	.97	.63
2. Directing	67.5°	80.20° (4.43°)	12.70°	.93	.79
3. Demanding‐strict	112.5°	102.19° (5.55°)	10.31°	.94	.77
4. Admonishing	157.5°	142.99° (5.52°)	14.51°	.92	.77
5. Ignoring‐resigning	202.5°	211.05° (6.61°)	8.55°	.95	.73
6. Indeterminate‐waiting	247.5°	248.53° (4.32°)	1.03°	.99	.82
7. Participating‐tolerating	292.5°	294.65° (4.44°)	2.15°	.99	.79
8. Understanding‐interested	337.5°	342.68° (8.92°)	5.18°	.97	.65

*Note*: Parameters were derived from the SPMC model with *m* = 2 Fourier series components. θ_target_ = Target scale position according to the theoretical model. $\hat{\theta }$ = Estimated scale position. *SE* = Standard error. Fisher's A* = 1 – $\left|\Delta \hat{\theta }\right|$/180 (Fisher et al., 1985). $\hat{h}$ = Estimated communality. $\hat{h}$ adheres to ${\hat{h}}_{i}=\sqrt{\frac{1}{\left(1+u_{i}\right)}}$, with *u*_*i*_ being the estimated unique variance of each octant indicator (Nagy et al., 2009).

The key structural components of our interpersonal teacher behaviour measure were invariant across girls and boys (Note S2 and Table S4). Additionally, regarding the student measures (math anxiety, perceived teacher‐expectation and self‐concept), we achieved metric invariance (Table S5). This measurement invariance serves as a prerequisite for comparing regression weights between groups (Chen, 2007).

Since student‐perceived teacher‐expectation and self‐concept were highly correlated (*r* = .84, Table 2), we employed one‐ and two‐factor CFAs to assess whether both constructs were empirically separable. Results revealed that the two‐factor model (*χ* ^2^ (19) = 50.73, *p* < .001, RMSEA = .048, CFI = .984, SRMR = .026) demonstrated a better fit to the data than the one‐factor solution (SB‐Δχ ^2^ = 125.37, Δdf = 1, *p* < .001). We therefore proceeded with the two‐factor solution.

**TABLE 2:** Means, standard deviations and correlations for all study variables.

Variable	*M* (*SD*)	ICC	1	2	3	4	5	6	7
1. Student math anxiety^a^	2.18 (.64)	.17							
2. Student self‐concept^a^	2.85 (.81)	.02	−.58***						
3. Student perceived teacher expectation^a^	2.96 (.62)	.03	−.50***	.84***					
4. Student age	10.07 (1.33)	.68	.04	−.14***	−.15***				
5. Student math competence	.57 (1.37)	.20	−.39***	.34***	.26***	.21***			
6. Student school satisfaction	2.91 (.77)	.08	−.33***	.25***	.33***	−.15***	−.04		
7. Teacher agency	.22 (1.31)	.23	.28***	−.27***	−.27***	−.09**	−.38***	−.13**	
8. Teacher communion	1.35 (.74)	.35	−.04	.03	.05	.15***	.05	.08*	.08*

*Note*: *M* and *SD* are used to represent mean and standard deviation, respectively.

Abbreviation: ICC, intraclass correlation coefficient.

^a^ Latent means were computed.

* *p* < .05.

** *p* < .01.

*** *p* < .001.

### Interpersonal teacher behaviour and math anxiety

Descriptive statistics for the full sample are provided in Table 2, while Table S2 presents the descriptive statistics separately for boys and girls. The ICC values of teacher agency (ICC = .23) and communion (ICC = .35) demonstrate moderate consistency in interpersonal teacher behaviour across different students within the same classroom.

Supporting Hypothesis 1, student math anxiety was most strongly associated with “demanding‐strict” interpersonal teacher behaviour (high agency combined with moderately low communion). This link was additionally supported by the correlation pattern in Figure S2. The standardized estimates (Table 3) indicated that, after controlling for student age, math competence and school satisfaction, student math anxiety was higher when teacher agency was higher (β = .11, *p* = .011). Figure 2 visualizes these relationships as a projection of math anxiety onto the circumplex. The summary vector for this association is located at 95.19° (“demanding‐strict”) with a vector length of VL = .10.

**TABLE 3:** Results from the SEM on student math anxiety and multiple‐group analysis on girls' and boys' math anxiety.

	Full sample math anxiety			Multiple‐group analysis					
				Girls' math anxiety			Boys' math anxiety		
	Est. (*SE*)	Std. Est	*p*	Est. (*SE*)	Std. Est	*p*	Est. (*SE*)	Std. Est	*p*
Math competence	−.17 (.02)	−.38	<.001***	−.10 (.03)	−.20	<.001***	−.16 (.03)	−.39	<.001***
Age	.05 (.02)	.10	.028*	.05 (.03)	.10	.131	.05 (.02)	.11	.058
School satisfaction	−.17 (.04)	−.20	<.001***	−.21 (.05)	−.24	<.001***	−.14 (.04)	−.20	<.001***
Agency	.06 (.02)	.11	.011*	.12 (.03)	.24	<.001***	.06 (.02)	.13	.003**
Communion	.00 (.03)	−.01	.891	−.14 (.05)	−.16	.007**	.03 (.04)	.03	.503
$R^{2}$	.23		<.001***	.26		<.001***	.25		<.001***

*Note*: *N* = 957, *n*_girls_ = 362, *n*_boys_ = 379. Model fit of the full sample analysis: *χ* ^2^ (53) = 172.93, *p* < .001, CFI = .950; RMSEA = .049; SRMR = .033 and multiple‐group analysis: *χ* ^2^ (120) = 255.21, *p* < .001, CFI = .937; RMSEA = .056; SRMR = .048.

* *p* < .05.

** *p* < .01.

*** *p* < .001.

**FIGURE 2:**
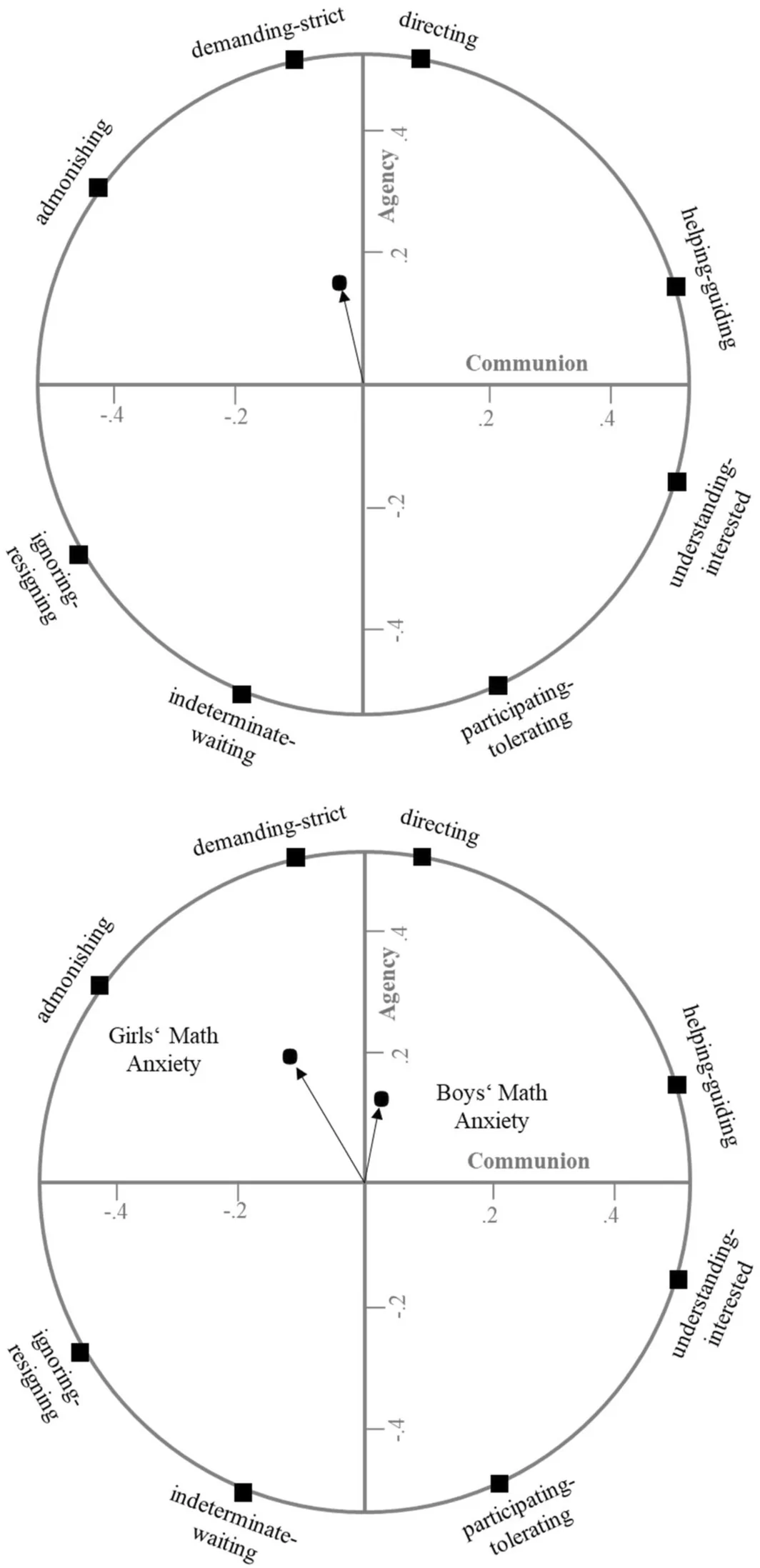
Profile plot for full‐sample (top) as well as girls' and boys' (bottom) math anxiety. The octant scale positions are estimated by the SPMC model.

Next, we explored gender differences. Multiple‐group analysis revealed a significant gender difference in the association with communion. Specifically, the math anxiety of girls was predominantly associated with the octant “admonishing”, placing the summary vector at 123.69° with VL = .18 (Figure 2). For boys, the summary vector pointed to 77.01° with VL = .13, indicating that boys' math anxiety was most strongly associated with the octants “demanding‐strict” and “directing”. Regarding the path weights for agency and communion, girls' math anxiety was positively associated with teacher agency (β = .24, *p* < .001) and negatively related to teacher communion (β = − .16, *p* = .007). For boys, math anxiety was also positively associated with teacher agency (β = .13, *p* = .003). However, deviating from the results for girls, boys' math anxiety was unrelated to teacher communion (β = .03, *p* = .503). Model comparison showed a significant gender difference in estimates for teacher communion (SB‐Δχ ^2^ = 5.16, Δdf = 1, *p* = .023, ΔCFI = .002) but not for teacher agency (SB‐Δχ ^2^ = 2.78, Δdf = 1, *p* = .095, ΔCFI = .000).

### Testing indirect associations with student teacher‐expectation and self‐concept

Finally, we investigated the indirect associations of interpersonal teacher behaviour and student math anxiety through student‐perceived teacher‐expectation (Model 2a) and student self‐concept (Model 2b). Further, in Model 2c, we included both student‐perceived teacher‐expectations and self‐concept simultaneously to account for their shared variance. Tables 4 and 5 display path estimates and estimates of indirect associations for the full sample. Teacher agency was indirectly linked with student math anxiety through student‐perceived teacher‐expectation, supporting Hypothesis 2a (Model 2a; β = .10, 95% CI = [.06; .15]). It was also indirectly linked with student self‐concept, supporting Hypothesis 3a (Model 2b; β = .12, 95% CI = [.08; .18]). Compared to Models 2a and 2b, the indirect associations through perceived teacher‐expectation were no longer statistically significant when both variables were included together (Model 2c). This outcome may stem from the substantial overlap between both mediators (*r* = .84, Table 2) and the limited incremental predictive power of perceived teacher‐expectation beyond self‐concept. Contrary to Hypotheses 2b and 3b, there were no indirect associations between student math anxiety and teacher communion.

**TABLE 4:** Results from the model of teacher agency and communion on student math anxiety via self‐concept and perceived teacher expectation.

Type	Est. (*SE*)	Std. Est	*p*
**Model 2a (via perceived teacher expectation)**			
*Component (a, b)*			
Agency → Perceived teacher expectation	−.13 (.02)	−.27	<.001***
Communion → Perceived teacher expectation	.03 (.04)	.03	.493
Perceived teacher expectation → Math anxiety	−.39 (.05)	−.38	<.001***
*Direct effects (c´)*			
Agency → Math anxiety	.03 (.02)	.06	.171
Communion → Math anxiety	.01 (.03)	.01	.823
*Model 2b (*via *self‐concept)*			
*Component (a, b)*			
Agency → Self‐concept	−.17 (.03)	−.27	<.001***
Communion → Self‐concept	.01 (.05)	.00	.917
Self‐concept → Math anxiety	−.36 (.04)	−.46	<.001***
*Direct effects (c´)*			
Agency → Math anxiety	.03 (.02)	.05	.207
Communion → Math anxiety	.00 (.03)	.00	.994
**Model 2c (via perceived teacher expectation & self‐concept)**			
*Component (a, b)*			
Agency → Perceived teacher expectation	−.13 (.02)	−.27	<.001***
Communion → Perceived teacher expectation	.03 (.04)	.03	.523
Perceived teacher expectation → Math anxiety	−.03 (.09)	−.03	.723
Agency → Self‐concept	−.17 (.03)	−.28	<.001***
Communion → Self‐concept	.00 (.03)	.00	.929
Self‐concept → Math anxiety	−.34 (.07)	−.44	<.001***
*Direct effects (c´)*			
Agency → Math anxiety	.02 (.02)	.06	.201
Communion → Math anxiety	.00 (.03)	.00	.997

*Note*: *N* = 957. Paths were controlled for student math competence, school satisfaction and age. We additionally specified covariances for: perceived teacher expectation with self‐concept, math competence with self‐concept, perceived teacher expectation age and agency, age with self‐concept and perceived teacher expectation. Model ‘perceived teacher expectation’: *χ* ^2^ (118) = 344.69, *p* < .001, RMSEA = .045, CFI = .944, SRMR = .055; Model ‘self‐concept’: *χ* ^2^ (87) = 247.11, *p* < .001, RMSEA = .044, CFI = .958, SRMR = .045; Model ‘perceived teacher expectation & self‐concept’: *χ* ^2^ (166) = 469.78, *p* < .001, RMSEA = .044, CFI = .948, SRMR = .055.

*** *p* < .001.

**TABLE 5:** Indirect effects from the model of teacher agency and communion on student math anxiety via self‐concept and perceived teacher expectation.

	Est. (*SE*)	Std. Est	Bc‐bootstrapped 95%‐CI
*Model 2a (via perceived teacher expectation)*			
Agency → Perceived teacher expectation → Math anxiety	.**04 (.01)**	.**10**	**[.06; .15]**
Communion → Perceived teacher expectation → Math anxiety	−.01 (.02)	−.01	[−.05; .02]
*Model 2b (*via *self‐concept)*			
Agency → Self‐concept→ Math anxiety	.**06 (.01)**	.**12**	**[.08; .18]**
Communion → Self‐concept → Math anxiety	.00 (.02)	.00	[−.04; .04]
*Model 2c (*via *perceived teacher expectation & self‐concept)*			
Agency → Perceived teacher expectation → Math anxiety	.00 (.01)	.00	[−.05; .05]
Communion → Perceived teacher expectation → Math anxiety	.00 (.00)	.00	[−.01; .01]
Agency → Self‐concept→ Math anxiety	.**07 (.02)**	.**13**	**[.08; .20]**
Communion → Self‐concept → Math anxiety	.00 (.02)	.00	[−.04; .04]

*Note*: We used bias‐corrected 20,000‐bootstrap analysis to estimate confidence intervals of the indirect effects (MacKinnon et al., 2004). Significant associations are bolded.

Regarding our gender specific analyses, Tables S6 and S7 display the path estimates and estimates of indirect associations from the multiple‐group analyses. The paths between teacher agency and communion, on the one hand, and girls' math anxiety, on the other, were indirectly associated through girls' perceived teacher‐expectation (for agency: β = .08, 95% CI = [.04; .15]; for communion: β = − .07, 95% CI = [−.14; −.03]) and through girls' self‐concept (for agency: β = .15, 95% CI = [.09; .22]; for communion: β = − .10, 95% CI = [−.15; −.04]), supporting Hypotheses 2a, 2b, 3a and 3b. Regarding boys, the relation between teacher agency and math anxiety was indirectly associated through perceived teacher‐expectation (β = .13, 95% CI = [.07; .20]) and through self‐concept (β = .12, 95% CI = [.06; .19]), supporting Hypotheses 2a and 3a. As in the full sample analyses, no indirect associations occurred between teacher communion and boys' math anxiety. When both mediators were included simultaneously (see Table S7), the indirect associations through perceived teacher‐expectation were substantially reduced. This attenuation again indicates shared variance between perceived teacher‐expectation and self‐concept.

## DISCUSSION

This study investigated the relationship between dyadic interpersonal teacher behaviour and student math anxiety from an integrative perspective based on interpersonal theory (Horowitz & Strack, 2011). Utilizing the interpersonal circumplex model (Gurtman & Balakrishnan, 1998), we simultaneously accounted for teacher agency and communion. This framework allowed us to examine how specific configurations of teacher behaviour relate to student math anxiety. Furthermore, we incorporated students' cognitive appraisals to shed light on potential mechanisms underlying these associations. Moving beyond previous studies (O'Hara et al., 2022), we investigated these dynamics at the level of individual student‐teacher dyads by employing a multi‐informant approach that combined teacher‐reported behaviour with student‐reported math anxiety, math self‐concept and perceived teacher expectations.

### What configuration of interpersonal teacher behaviour relates to student math anxiety and cognitive appraisals?

Consistent with our expectation, “demanding‐strict” interpersonal teacher behaviour showed the strongest association with math anxiety in primary school students. This aligns with previous research, which linked classroom‐level math anxiety to teacher behaviours such as fear appeals, punishment or competition (Frenzel et al., 2007; Putwain & Symes, 2011; Westphal et al., 2018). One possible explanation for the association between “demanding‐strict” teacher behaviour and student math anxiety is that strong teacher agency combined with weak communion is characteristic of a negative, maladaptive learning environment (Goetz et al., 2021; Zee & Roorda, 2018). In such environments, students may lack emotional reassurance while their sense of autonomy and competence is severely diminished (Aelterman & Vansteenkiste, 2023). Alternatively, this relationship could reflect a reciprocal dyadic dynamic. Specifically, math‐anxious, passive students who exhibit low agency may elicit highly agentic responses from teachers (Pennings et al., 2018). This interpretation aligns with the complementarity principle of interpersonal theory (Sadler et al., 2009), which posits that an actor's low agency naturally pulls for high agency in their interaction partner.

The strong association between math anxiety and teacher behaviour high in agency and low in communion implies that low math anxiety is linked to teacher behaviour low in agency and high in communion (see also Figure S2). This directly follows from the circular structure of the correlation profile. Consequently, “understanding‐interested” and “participating‐tolerating” teacher behaviours within the dyad co‐occur with higher levels of student agency. This, in turn, is tied to increased feelings of autonomy, competence and positive affect in math. Our findings regarding indirect associations further support this interpretation. Elevated teacher agency is often met with reduced student agency (Sadler et al., 2009) and cooperation (Van Vemde et al., 2022). Consequently, highly directive teacher behaviour may limit students' opportunities to independently engage with the demands of math lessons. Failing to meet these academic requirements is subsequently associated with lower math self‐concept and higher math anxiety (Goetz et al., 2010, 2020). Additionally, students may interpret close teacher guidance as a sign of low expectations regarding their math competence (Gentrup et al., 2020), which further relates to higher anxiety levels (Hollenstein et al., 2023).

### The role of gender

Boys' math anxiety was uniquely related to teacher agency, showing the strongest association for “demanding‐strict” interpersonal teacher behaviours. In contrast, girls' math anxiety was related to both dimensions, showing a positive association with teacher agency and a negative association with teacher communion (“admonishing” interpersonal teacher behaviour). These findings suggest that low teacher communion may be particularly relevant for the experience of math anxiety in girls. This aligns with research on student‐teacher relationship quality, indicating that girls' math achievement, more than boys', is associated with a close and non‐conflictual relationship with the teacher (McCormick & O'Connor, 2015; Valiente et al., 2019). Consequently, girls' emotional experience in math may be more sensitive to the teacher's interpersonal behaviour.

Girls' heightened sensitivity may be discussed in light of pervasive gender stereotypes that characterize math as a male domain (Nosek et al., 2002). These stereotypes can cause girls to feel more uncertain about their own mathematical abilities (Henschel et al., 2023). As a result, they may monitor teachers' interpersonal behaviour more closely for signals regarding their own academic competence. Such dynamics mirror self‐fulfilling prophecy mechanisms, where teacher expectations can shape student outcomes through differential treatment (Rosenthal & Jacobson, 1968).

When teachers hold lower expectations for girls in math (Gentrup & Rjosk, 2018; Siems‐Muntoni et al., 2025), these beliefs may be mirrored in less supportive interpersonal behaviours. Such behaviour may, in turn, co‐occur with lower academic confidence among girls and reinforce existing gender stereotypes. Conversely, an “optimistic teacher‐student relationship” (Steele, 1997, p. 624)—one in which girls are actively encouraged and reassured of their competence—may mitigate the learning impairment induced by stereotypes (Liu et al., 2021). Consistent with this interpretation, our results on the indirect associations between interpersonal teacher behaviour and math anxiety indicate that dyadic interactions with a girl characterized by warm, affectionate teacher behaviour (high communion) and autonomy‐granting practices (low agency) relate to greater confidence in her own abilities (self‐concept) and a stronger belief that the teacher views her as competent (teacher‐expectations).

In contrast to our findings for girls, teacher communion was related neither to boys' perceived teacher expectations nor to their self‐concept. Research consistently shows that boys tend to have less close and more conflictual relationships with teachers (Hajovsky et al., 2017; Kang et al., 2023) and perceive lower levels of emotional support compared to girls (Arens & Niepel, 2019; Lietaert et al., 2015). Consequently, boys appear less likely than girls to infer their teacher's expectations from the teacher's behaviour and to base their self‐concept on that behaviour (Buckingham et al., 2012; Crocker et al., 2006).

### Limitations and directions for future research

Several limitations of our study must be noted. First, we used a cross‐sectional design. Since dyadic interpersonal behaviour is in fact reciprocal (Sadler et al., 2009), it remains to be determined whether teacher behaviour constitutes a proactive antecedent of or a reactive adaptation to student math anxiety. For example, teachers may exhibit higher communion and lower agency toward anxious students as a compensatory response, rather than their behaviour directly predicting student anxiety. Consequently, the role of teachers may be less direct, and our findings should be interpreted as correlational associations rather than evidence of causal teacher effects. To address this, future research should employ longitudinal or experimental designs to examine the potentially bidirectional influences between interpersonal teacher behaviour and student math anxiety more robustly. Longitudinal studies tracking student‐teacher dyads over time would help untangle how these reciprocal processes unfold. Experimental studies could utilize video‐based or structured role‐plays to systematically manipulate specific teacher behaviours—such as varying warmth (communion) or instructional guidance (agency). Testing subsequent changes in students' math anxiety within such designs would clarify whether higher communion and lower agency function as protective factors or predominantly reflect teachers' adaptive responses to student distress.

A second limitation is that primary school teachers in Germany typically teach multiple subjects rather than specializing exclusively in math. When describing their interpersonal behaviour toward individual students, teachers likely reflected on their general behaviour rather than subject‐specific interactions. While it is improbable that in primary school a teacher's behaviour toward a specific student varies significantly across subjects, such variation cannot be entirely ruled out. For instance, a student might exhibit different behaviours in different subjects, thereby eliciting distinct reactions from the teacher. Furthermore, our data do not allow us to determine whether low teacher communion and high agency are linked to subject‐specific anxiety in areas other than math. Consequently, this limitation reduces the specificity of our conclusions regarding math anxiety. Although we tried to mitigate this issue by controlling for general school satisfaction, it remains a task for future research to assess teacher interpersonal behaviour and student anxiety across various school subjects simultaneously. Such studies could examine whether teacher communion and agency operate differently in math compared to language or science classes and whether math anxiety is more strongly linked to teacher behaviour during math instruction than to their interpersonal behaviour in general.

A third limitation concerns the gender distribution of our teacher sample, which was predominantly female (76%). Although this distribution aligns with the gender distribution among primary school teachers in Germany (Statistisches Bundesamt [German Federal Statistical Office], 2023), it restricted our ability to investigate whether the interpersonal behaviour of male teachers would yield different outcomes. For instance, past research suggests that boys are less likely to form close attachment relationships with female teachers compared to male teachers (Ahnert et al., 2006); consequently, boys' math anxiety might be less sensitive to variations in female teachers' communion. Conversely, it is conceivable that an indirect relationship between teacher communion and student math anxiety via students' cognitive appraisals would emerge for boys when taught by male teachers (see Wolter et al., 2014). Our findings might therefore be specific to classrooms with female instructors, meaning that generalizing these results to male teachers should be done with caution. Future studies should explicitly examine whether the links between teacher communion and agency and students' cognitive appraisals and emotions at school differ depending on teacher gender, student gender or teacher–student gender match.

A fourth limitation is the strong intercorrelation between student‐perceived teacher‐expectation and self‐concept. This multicollinearity likely stems from assessing both constructs at the student level using similarly worded items. Such a measurement overlap may have inflated the association between the two variables and reduced their unique predictive contributions when modelled together. Consequently, the relative strengths of the indirect links of perceived teacher‐expectation and self‐concept to math anxiety should be interpreted with caution. Future studies should address this issue by employing more distinct item wording, multi‐informant approaches or longitudinal designs that permit a clearer conceptual and temporal separation.

### Conclusion and practical implications

The findings of this study underscore the critical role of interpersonal teacher behaviour in relation to math anxiety in primary school students (O'Hara et al., 2022). Specifically, we demonstrate that dyadic interpersonal teacher behaviour is systematically linked to students' math anxiety and associated cognitive appraisals (Mainhard et al., 2018; Pekrun, 2006). Using the interpersonal circumplex framework (Gurtman & Balakrishnan, 1998), we extend previous findings by showing that focusing solely on either the socio‐emotional (communion) or instructional (agency) dimension of teacher behaviour is insufficient. Instead, teacher behaviour is best understood as an integrated blend of both agency and communion. Consequently, teacher professional development should focus on cultivating “understanding‐interested” and “participating‐tolerating” behaviours in dyadic interactions. These interaction patterns—marked by high communion and moderate‐to‐low agency—relate to lower levels of math anxiety and characterize learning environments that are both warm and supportive of student autonomy.

## AUTHOR CONTRIBUTIONS

**M. Frühauf:** Conceptualization; writing – original draft; methodology; visualization; formal analysis; data curation; investigation. **J. M. Etzel:** Methodology; formal analysis; writing – review and editing. **B. Hannover:** Conceptualization; funding acquisition; supervision; writing – review and editing.

## CONFLICT OF INTEREST STATEMENT

The authors declare no conflicts of interest.

## Supporting information


**Note S1.** Technical Details and Model Specification of the Circular Stochastic Process Model.
**Note S2**. Additional Information on Standard Error Estimation.
**Table S1**. Octant Descriptions of the Circumplex Model on Dyadic Teacher Interpersonal Behaviour.
**Table S2**. Means, Standard deviations and Correlations for Girls (Above the Diagonal) and Boys (Below the Diagonal).
**Table S3**. Model Fit Comparison of the SPMC Models.
**Table S4**. Invariance Test of the SPMC Model for Girls and Boys.
**Table S5**. Invariance Test of Math Anxiety, Self‐Concept and Perceived Teacher Expectation for Girls and Boys.
**Table S6**. Results from Multiple‐Group Model of Teacher Agency and Communion on Student Math Anxiety via Self‐Concept and Perceived Teacher Expectation.
**Table S7**. Indirect Effects from Multiple‐Group Model of Teacher Agency and Communion on Student Math Anxiety via Self‐Concept and Perceived Teacher Expectation.
**Figure S1**. Measurement Model of Student Math Anxiety, Self‐Concept and Perceived Teacher Expectation.
**Figure S2**. Correlational Patterns between Interpersonal Teacher Behaviour Octants and Student Math Anxiety.

## Data Availability

The study materials, analysis syntaxes and data are available at https://osf.io/pcfbk.

## Footnotes

^1^
The math anxiety scale originally differentiates between cognitive (“worry”) and affective (“nervousness”) components of math anxiety. Confirmatory factor analysis (CFA) revealed that the two‐factor solution (*χ* ^2^ (16) = 81.50, *p* < .001, RMSEA = .076, CFI = .965, SRMR = .031) demonstrated a poorer model fit compared to the one‐factor model (*χ* ^2^ (12) = 12.92, *p* = .375, RMSEA = .010, CFI = 1.00, SRMR = .013; SB‐Δχ ^2^ = 69.12, Δdf = 4, *p* < .001, ΔCFI = .035). Hence, we used a general factor of math anxiety for all subsequent analyses.
