# Supplementary material for: How teacher behaviour in student‐teacher dyads relates to boys' and girls' mathematics anxiety. An investigation of interpersonal profiles
Source: Br J Educ Psychol. 2026 Jun 26;96(4):1593–614. doi: 10.1111/bjep.70104 (PMC13752655; doi:10.1111/bjep.70104)
Supplement: Supplementary file 1 [file BJEP-96-1593-s001.docx]

**Supplementary Material for the Article:**

**How Teacher Behavior in Student-Teacher Dyads Relates to Boys' and Girls' Mathematics Anxiety.**

**An Investigation of Interpersonal Profiles**

**Note S1**

*Technical Details and Model Specification of the Circular Stochastic Process Model*

The circular stochastic process model (SPMC; Browne, 1992) tests whether correlation pattern of the circumplex indicators (here: the eight octant scales) adheres to the circumplex structure. The model assumes that each indicator variableconsists of a common, $c_{i}$¸and a unique part, $u_{i}$. Importantly, the SPMC models the (quasi-)circumplex structure at the level of the correlations between the common scores of two scales *j* and *k*. In other words, it assumes a latent (quasi-)circumplex structure, thus accounting for the fallible nature of observed scale scores (i.e., measurement error). In this model, the correlation between the common scores of any two indicator variables are linked to the smallest separating angle between their locations circumplex (for instance, the octant scales "admonishing" and "demanding-strict" should be separated by approx. 157.5°- 112.5° = 45°). To flexibly model this correlational pattern, Browne (1992) utilizes a Fourier series approximation, where the observed correlation of any two indicators *j* and *k* is defined by:

$$r_{j, k}= \hat{h_{j}}\left\{ \hat{\beta_{0}}+ \sum_{m=1}^{M} \hat{\beta_{m}}\cos\left. \left[ m\left. \left( \hat{\theta_{j}}- \hat{\theta_{k}} \right. \right) \right. \right] \right\}\hat{h_{k}}$$

with *m* describing the number of Fourier components (with *m* = 1 components, the correlation function reduces to a regular cosine function) and the estimated $\hat{\beta}$-parameters define the level and shape of the (estimated) correlation function. The $\hat{\beta}$-parameters must adhere to $\beta_{0}= 1- \sum_{m=1}^{M} \beta_{m}$ and are all constrained to be larger than 0. $\hat{\theta_{j}}$ and $\hat{\theta_{k}}$ are the estimated angular positions of the octant scales *j* and *k* in the latent circumplex. The $\hat{h_{j}}$ and $\hat{h_{k}}$ describe the estimated scale communalities indicating how strongly the octant indicator relates to the latent circumplex. For more information and technical details see Browne (1992), Nagy et al. (2010, 2019) and Etzel et al. (2024).

**Note S2**

*Invariance Test of the SPMC and Student Measures*

Since we wanted to investigate gender differences in the association of student math anxiety with the teacher behavior circumplex, we tested whether our circumplex model was invariant across student gender groups to rule out that gender differences were due to differences in the circular structure of the teacher behavior measure. We followed the established procedure (Etzel et al., 2021; Nagy et al., 2010) by estimating a multigroup SPMC using the GROUPING command in Mplus and stepwise constraining (a) equal beta-parameters (e.g., constraining the shape of the correlation function to be equal across gender), (b) equal theta-parameters (e.g., constraining octant scales to have the same angular location across gender), (c) equal uniquenesses (e.g., constraining the unique variances for octant scales across gender), and (d) equal scaling constants (e.g., constraining common factor to enter with the same weight into the observed octant variable for both gender groups). Additionally, we tested measurement invariance as a prerequisite to ensure comparability (Chen, 2007) of the student measures (math anxiety, self-concept, and perceived teacher expectation). Following van de Schoot et al. (2012) by estimating model parameters simultaneously for both gender groups and stepwise testing (a) configural invariance (e.g., constraining an equal factor pattern across gender), (b) metric invariance (e.g., constraining invariant factor loadings across gender), (c) scalar invariance (e.g., constraining equal item intercepts across gender), and (d) strict invariance (e.g., constraining equal item uniqueness across gender). According to Cheung and Rensvold (2002), $\left| \Delta CFI \right|$ < .010 indicates measurement invariance. We applied χ^2^-difference test with Satorra-Bentler scaling correction (Satorra & Bentler, 2010) for model fit comparison. The results of the invariance tests can be found in Table S3 for the SPMC and Table S4 for the student measures.

**Note S2**

*Additional Information on Standard Error Estimation*

For some estimated models, the M*plus* software produced a warning stating that the standard errors of the models may not be trustworthy due to the models having more parameters than the number of clusters. In such cases, we conducted two robustness checks by (1) estimating standard errors without cluster correction, and (2) estimating standard errors using 20,000 bootstraps that is recommended in case of small cluster sizes (Cameron et al., 2008). Both models were compared to the model with cluster-robust SE (TYPE = COMPLEX). In all cases, only small change in standard errors occurred that were not relevant to the central results of the study and therefore, we proceeded with the initial models. All robustness check models can be found in the OSF: <https://osf.io/pcfbk/?view_only=659c3ab0dd4e450e8f70221d12dd18f9>.

**Table S1**

*Octant Descriptions of the Circumplex Model on Dyadic Teacher Interpersonal Behavior*

| Octant Label | Theoretical Position in the Circumplex Structure | Description | Sample Item |
| --- | --- | --- | --- |
| Helping-guiding | 22.5°  [0°- 45°] | In a warm, affectionate, and caring manner, the teacher provides clear structures for the child to orientate themselves. | *If [unique identifier] cannot yet solve a task correctly, I help them to identify any misconceptions and thus to find the solution.* |
| Directing | 67.5°  [45°- 90°] | In an appreciative manner, the teacher gives the child very precise instructions, monitors, and, if necessary, corrects each of their behavior. | *When I give assignments, I always check that [unique identifier] understands what they need to do.* |
| Demanding-strict | 112.5°  [90°- 135°] | The teacher does not address the child's interests or perspective in particular but rather gives very precise instructions, monitors, and, if necessary, corrects any of their behaviors. | *I require [unique identifier] to complete assigned tasks exactly as given.* |
| Admonishing | 157.5°  [135°- 180°] | The teacher has a distant, dismissive attitude towards the child and tries to guide and control the child's behavior through frequent admonitions. | *I do not give [unique identifier] too much leeway so that they do not ruin my lesson plan.* |
| Ignoring-resigning | 202.5°  [180°- 225°] | The teacher has a distant, dismissive attitude towards the child and turns to them only when it cannot be avoided. | *I teach the learning content according to plan, without letting [unique identifier] get in the way.* |
| Indeterminate-waiting | 247.5°  [225°- 270°] | The teacher is not concerned with the interests or the perspective of the child in particular but simply lets the child have their way. | *I just let [unique identifier] run along in class.* |
| Participating-tolerating | 292.5°  [270°- 315°] | In an appreciative manner, the teacher signals to the child that he or she can pursue their own interests and then simply lets them do so. | *I give [unique identifier] in particular a lot of freedom when learning.* |
| Understanding-interested | 337.5°  [315°- 360°] | In a cordial, attentive, and caring manner, the teacher creates freedom for the child to identify their interests. | *I listen particularly to [unique identifier] always patiently and interested when they contribute something in class.* |

*Note.* Octant descriptions are translated and adopted from (Hannover et al., 2022). The full item set can be found at Kreutzmann et al. (2024). To maintain anonymity, each student was assigned a unique identifier (tree name).

**Table S2**

*Means, Standard deviations, and Correlations for Girls (Above the Diagonal) and Boys (Below the Diagonal)*

| Variable | *M_girls_*  (*SD*) | *M_boys_*  (*SD*) | 1 | 2 | 3 | 4 | 5 | 6 | 7 | 8 |
| --- | --- | --- | --- | --- | --- | --- | --- | --- | --- | --- |
|  |  |  |  |  |  |  |  |  |  |  |
| 1. Student math anxiety^a^ | 2.36  (0.68) | 1.99  (0.53) |  | -.59^***^ | -.49^***^ | .05 | -.32^***^ | -.31^***^ | .34^***^ | -.20^***^ |
| 2. Student self-concept^a^ | 2.65  (0.79) | 3.08  (0.76) | -.50^***^ |  | .80^***^ | -.06 | .38^***^ | .30^***^ | -.33^***^ | .21^***^ |
| 3. Student perceived teacher expectation^a^ | 2.87  (0.65) | 3.06  (0.57) | -.49^***^ | .86^***^ |  | -.12^*^ | .30^***^ | .39^***^ | -.26^***^ | .23^***^ |
| 4. Student age | 10.06  (1.31) | 10.10  (1.35) | .05 | -.21^***^ | -.17^**^ |  | .18^**^ | -.11* | -.08 | -.08 |
| 5. Student math competence | 0.40  (1.27) | 0.79  (1.43) | -.40^***^ | .26^***^ | .20^***^ | .23^***^ |  | .04 | -.44^***^ | .11^*^ |
| 6. Student school satisfaction | 2.96  (0.74) | 2.88  (0.79) | -.21^***^ | .26^***^ | .32^***^ | -.19^***^ | -.08 |  | -.08 | .11^*^ |
| 7. Teacher agency | 0.05  (1.31) | 0.31  (1.32) | .30^***^ | -.30^**^ | -.34^***^ | -.11^*^ | -.39^***^ | -.16^**^ |  | .00 |
| 8. Teacher communion | 1.53  (0.70) | 1.23  (0.69) | .00 | -.04 | -.02 | .18^***^ | .08 | .01 | -.15^**^ |  |
|  |  |  |  |  |  |  |  |  |  |  |

*Note.* *M* and *SD* are used to represent mean and standard deviation, respectively. ^a^Latent means were computed.

^*^ *p* < .05, ^**^ *p* < .01, ^***^ *p* < .001.

**Table S3**

*Model Fit Comparison of the SPMC Models*

|  |  | Goodness of Fit | | | | | |  |
| --- | --- | --- | --- | --- | --- | --- | --- | --- |
| Number of  Fourier components |  | $\chi$^2^ | df | *p* | RMSEA | CFI | SRMR |  |
| *m* = 1 |  | 43.09 | 12 | < .001 | .052  [.036, .070] | .940 | .040 |  |
| *m* = 2 |  | 32.77 | 11 | < .001 | .046  [.028, .064] | .958 | .049 |  |
| *m* = 3 |  | 30.06 | 10 | < .001 | .046  [.028, .065] | .961 | .062 |  |

*Note.* RMSEA = Root-Mean-Square Error of Approximation; CFI = Comparative Fit Index; SRMR = Standardized Root Mean Square Residuals.

**Table S4**

*Invariance Test of the SPMC Model for Girls and Boys*

|  |  | Goodness of fit | | | | | |  | Model comparison | | | |
| --- | --- | --- | --- | --- | --- | --- | --- | --- | --- | --- | --- | --- |
| Model |  | $\chi$^2^ | df | *p* | RMSEA | CFI | SRMR |  | SB-$\Delta\chi$^2^ | $\Delta df$ | *p* | $\Delta CFI$ |
| Unconstrained |  | 47.92 | 22 | .001 | .057  [.035, .079] | .960 | .048 |  | - | - | - | - |
| Equal betas |  | 47.83 | 24 | .003 | .052  [.030, .073] | .963 | .047 |  | 0.26 | 2 | .878 | .003 |
| Equal theta |  | 54.97 | 31 | .005 | .046  [.025, .065] | .963 | .053 |  | 5.77 | 7 | .567 | .000 |
| Equal uniquenesses |  | 62.13 | 39 | .011 | .040  [.020, .058] | .964 | .059 |  | 6.56 | 8 | .585 | .001 |
| Equal scaling |  | 68.83 | 47 | .021 | .036  [.014, 0.053] | .966 | .074 |  | 5.13 | 8 | .744 | .002 |

*Note.* $\chi$^2^ difference tests were adjusted using Satorra-Bentler scaling correction (SB; Santorra & Bentler, 2010). RMSEA = Root-Mean-Square Error of Approximation; CFI = Comparative Fit Index; SRMR = Standardized Root Mean Square Residuals.

**Table S5**

*Invariance Test of Math Anxiety, Self-Concept, and Perceived Teacher Expectation for Girls and Boys*

|  |  | Goodness of Fit | | | | | |  | Model Comparison | | | |
| --- | --- | --- | --- | --- | --- | --- | --- | --- | --- | --- | --- | --- |
| Model |  | $\chi$^2^ | df | *p* | RMSEA | CFI | SRMR |  | SB-$\Delta\chi$^2^ | $\Delta df$ | *p* | $\Delta CFI$ |
| Configural |  | 363.71 | 198 | < .001 | .048  [.040, .056] | .946 | .041 |  | - | - | - | - |
| Metric |  | 385.60 | 214 | < .001 | .047  [.040, .055] | .963 | .056 |  | 23.06 | 16 | .112 | -.017 |
| Scalar |  | 491.53 | 230 | < .001 | .056  [.049, .063] | .943 | .089 |  | 159.85 | 16 | < .001 | .020 |
| Strict |  | 506.14 | 246 | < .001 | .054  [.048, .061] | .943 | .094 |  | 19.40 | 16 | .248 | .000 |

*Note.* $\chi$^2^ difference tests were adjusted using Satorra-Bentler scaling correction (SB; Santorra & Bentler, 2010). RMSEA = Root-Mean-Square Error of Approximation; CFI = Comparative Fit Index; SRMR = Standardized Root Mean Square Residuals.

**Table S6**

*Results from Multiple-Group Model of Teacher Agency and Communion on Student Math Anxiety via Self-Concept and Perceived Teacher Expectation*

|  | *Girls* | | |  | *Boys* | | |
| --- | --- | --- | --- | --- | --- | --- | --- |
| Type | *Est. (SE)* | *Std. Est* | *p* |  | *Est. (SE)* | *Std. Est* | *p* |
| *Model 2a (via perceived teacher expectation)* | | | | | | | |
| *Component (a, b)* |  |  |  |  |  |  |  |
| Agency → Perceived teacher expectation | -0.12 (0.03) | -.27 | < .001^***^ |  | -0.16 (0.03) | -.35 | < .001^***^ |
| Communion → Perceived teacher expectation | 0.19 (0.05) | .23 | < .001^***^ |  | 0.07 (0.05) | -.08 | .177 |
| Perceived teacher expectation → Math anxiety | -0.34 (0.08) | -.32 | < .001^***^ |  | -0.34 (0.07) | -.37 | < .001^***^ |
| *Direct Effects (c´)* |  |  |  |  |  |  |  |
| Agency → Math anxiety | 0.09 (0.03) | .20 | .002^**^ |  | 0.02 (0.02) | .05 | .281 |
| Communion → Math anxiety | -0.09 (0.06) | -.10 | .114 |  | 0.02 (0.04) | .02 | .690 |
| *Model 2b (via self-concept)* | | | | | | | |
| *Component (a, b)* |  |  |  |  |  |  |  |
| Agency → Self-concept | -0.20 (0.04) | -.32 | < .001^***^ |  | -0.19 (0.04) | -.32 | < .001^***^ |
| Communion → Self-concept | 0.25 (0.06) | .22 | < .001^***^ |  | -0.10 (0.07) | -.09 | .161 |
| Self-concept → Math anxiety | -0.35 (0.05) | -.45 | < .001^***^ |  | -0.27 (0.05) | -.37 | < .001^***^ |
| *Direct Effects (c´)* |  |  |  |  |  |  |  |
| Agency → Math anxiety | 0.07 (0.03) | .16 | .014^*^ |  | 0.03 (0.02) | .06 | .130 |
| Communion → Math anxiety | -0.07 (0.05) | -.08 | .140 |  | 0.01 (0.04) | .02 | .742 |
| *Model 2c (via perceived teacher expectation & self-concept)* | | | | | | | |
| *Component (a, b)* |  |  |  |  |  |  |  |
| Agency → Perceived teacher expectation | -0.13 (0.03) | -.29 | < .001^***^ |  | -0.17 (0.03) | -.36 | < .001^***^ |
| Communion → Perceived teacher expectation | 0.17 (0.05) | .20 | < .001^***^ |  | -0.06 (0.05) | -.07 | .166 |
| Perceived teacher expectation → Math anxiety | 0.12 (0.14) | .11 | .369 |  | -0.20 (0.12) | -.22 | .081 |
| Agency → Self-concept | -0.21 (0.04) | -.34 | < .001^***^ |  | -0.20 (0.03) | -.34 | < .001^***^ |
| Communion → Self-concept | 0.24 (0.07) | .21 | < .001^***^ |  | -0.10 (0.07) | -.09 | .140 |
| Self-concept → Math anxiety | -0.41 (0.09) | -.52 | < .001^***^ |  | -0.14 (0.09) | -.18 | .159 |
| *Direct Effects (c´)* |  |  |  |  |  |  |  |
| Agency → Math anxiety | 0.08 (0.03) | .17 | .009^**^ |  | 0.02 (0.02) | .05 | .242 |
| Communion → Math anxiety | -0.07 (0.05) | -.08 | .152 |  | 0.02 (0.04) | .03 | .580 |

*Note. N* = 741, *n_girls_* = 362, *n_boys_* = 379. Paths were controlled for student math competence, school satisfaction and age. We additionally specified covariances for: perceived teacher expectation with self-concept, math competence with self-concept, perceived teacher expectation age and agency, age with self-concept and perceived teacher expectation. Model perceived teacher expectation: χ2 (258) = 506.76, *p* < .001, RMSEA = .051, CFI = .934, SRMR = .066; Model self-concept: χ2 (192) = 364.09, *p* < .001, RMSEA = .049, CFI = .951, SRMR = .055; Model perceived teacher expectation & self-concept: χ2 (366) = 719.54, *p* < .001, RMSEA = .053, CFI = .936, SRMR = .073.

^*^ *p* < .05, ^**^ *p* < .01, ^***^ *p* < .001.

**Table S7**

*Indirect Effects from Multiple-Group Model of Teacher Agency and Communion on Student Math Anxiety via Self-Concept and Perceived Teacher Expectation*

|  | *Girls* | | |  | *Boys* | | |
| --- | --- | --- | --- | --- | --- | --- | --- |
|  | *Est. (SE)* | *Std. Est* | Bc-Bootstrapped 95% CI |  | *Est. (SE)* | *Std. Est* | Bc-Bootstrapped 95% CI |
| *Model 2a (via perceived teacher expectation)* | | | | | | | |
| Agency → Perceived teacher expectation → Math anxiety | **0.04 (0.01)** | **.08** | **[0.04; 0.15]** |  | **0.06 (0.02)** | **.13** | **[0.07; 0.20]** |
| Communion → Perceived teacher expectation → Math anxiety | **-0.06 (0.02)** | **-.07** | **[-0.14; -0.03]** |  | 0.02 (0.02) | .03 | [-0.01; 0.08] |
| *Model 2b (via self-concept)* | | | | | | | |
| Agency → Self-concept→ Math anxiety | **0.07 (0.02)** | **.15** | **[0.09; 0.22]** |  | **0.05 (0.02)** | **.12** | **[0.06; 0.19]** |
| Communion → Self-concept → Math anxiety | **-0.09 (0.03)** | **-.10** | **[-0.17; -0.05]** |  | 0.03 (0.02) | .03 | [-0.01; 0.09] |
| *Model 2c (via perceived teacher expectation & self-concept)* | | | | | | | |
| Agency → Perceived teacher expectation → Math anxiety | -0.01 (0.02) | -.03 | [-0.12; 0.03] |  | 0.03 (0.02) | .08 | [-0.02; 0.17] |
| Communion → Perceived teacher expectation → Math anxiety | 0.02 (0.03) | .02 | [-0.02; 0.10] |  | 0.01 (0.02) | .02 | [-0.004; 0.08] |
| Agency → Self-concept→ Math anxiety | **0.09 (0.03)** | **.18** | **[0.09; 0.30]** |  | 0.03 (0.02) | .06 | [-0.03; 0.18] |
| Communion → Self-concept → Math anxiety | **-0.10 (0.04)** | **-.11** | **[-0.22; -0.05]** |  | 0.01 (0.02) | .02 | [-0.01; 0.08] |

*Note.* We used bias-corrected 20,000-bootstrap analysis to estimate confidence intervals of the indirect effects. Significant associations are bolded.

**Figure S1**

*Measurement Model of Student Math Anxiety, Self-Concept, and Perceived Teacher Expectation*

*Note*. For model estimation, we freed few and only theoretically plausible parameters (allowing errors for semantically similar items within one construct to covary) according to the modification indices (Sörbom, 1989). Model fit: χ2 (99) = 272.10, *p* < .001, RMSEA = .049, CFI = .964, SRMR = .034.

**Figure S2**

*Correlational Patterns between Interpersonal Teacher Behavior Octants and Student Math Anxiety*

*Note.* 1. helping- guiding, 2. directing, 3. demanding- strict, 4. admonishing, 5. ignoring- resigning, 6. indeterminate- waiting, 7. participating- tolerating, 8. understanding- interested.

**References**

Browne, M. W. (1992). Circumplex models for correlation matrices. *Psychometrika*, *57*(4), 469–497. https://doi.org/10.1007/BF02294416

Cameron, A. C., Gelbach, J. B., & Miller, D. L. (2008). Bootstrap-based improvements for inference with clustered errors. *Review of Economics and Statistics*, *90*(3), 414–427. https://doi.org/10.1162/rest.90.3.414

Chen, F. F. (2007). Sensitivity of goodness of fit indexes to lack of measurement invariance. *Structural Equation Modeling: A Multidisciplinary Journal*, *14*(3), 464–504. https://doi.org/10.1080/10705510701301834

Cheung, G. W., & Rensvold, R. B. (2002). Evaluating goodness-of-fit indexes for testing measurement invariance. *Structural Equation Modeling: A Multidisciplinary Journal*, *9*(2), 233–255. https://doi.org/10.1207/S15328007SEM0902_5

Etzel, J. M., Holland, J., & Nagy, G. (2021). The internal and external validity of the latent vocational interest circumplex: Structure, relationships with self-concepts, and robustness against item-order effects. *Journal of Vocational Behavior*, *124*, 103520. https://doi.org/10.1016/j.jvb.2020.103520

Etzel, J. M., Wille, B., De Fruyt, F., & Nagy, G. (2024). *Revisiting the relationship between personality traits and vocational interests from a profile-based perspective: Research synthesis and new applications*. https://doi.org/10.31234/osf.io/sm6j9

Hannover, B., Koeppen, K., & Kreutzmann, M. (2022). Die Qualität des Lehrkraftverhaltens in Lehrkraft-Kind-Dyaden: Unterschiede zwischen Kindern in Abhängigkeit ihrer Noten, ihres Geschlechts, ihrer sprachlichen Herkunft und eines sonderpädagogischen Förderbedarfs [Agency and communion of teacher behavior in dyadic interactions with primary school children. Differences between children depending on their grades, gender, linguistic background, and special educational needs.]. *Zeitschrift für Pädagogische Psychologie*, *36*(1–2), 65–84. https://doi.org/10.1024/1010-0652/a000327

Kreutzmann, M., Frühauf, M., Hannover, B., & Koeppen, K. (2024). Communal and agentic teacher behavior in teacher-child dyads. A new self-report scale based on a circumplex approach. *Frontiers in Education*, *9*, 1397936. https://doi.org/10.3389/feduc.2024.1397936

MacKinnon, D. P., Lockwood, C. M., & Williams, J. (2004). Confidence limits for the indirect effect: Distribution of the product and resampling methods. *Multivariate Behavioral Research*, *39*(1), 99–128. https://doi.org/10.1207/s15327906mbr3901_4

Nagy, G., Trautwein, U., & Lüdtke, O. (2010). The structure of vocational interests in Germany: Different methodologies, different conclusions. *Journal of Vocational Behavior*, *76*(2), 153–169. https://doi.org/10.1016/j.jvb.2007.07.002

Satorra, A., & Bentler, P. M. (2010). Ensuring positiveness of the scaled difference Chi-square test statistic. *Psychometrika*, *75*(2), 243–248. https://doi.org/10.1007/s11336-009-9135-y

Sörbom, D. (1989). Model modification. *Psychometrika*, *54*(3), 371–384.

van de Schoot, R., Lugtig, P., & Hox, J. (2012). A checklist for testing measurement invariance. *European Journal of Developmental Psychology*, *9*(4), 486–492. https://doi.org/10.1080/17405629.2012.686740
